# Recognizing Psychosis in Autism Spectrum Disorder

**DOI:** 10.3389/fpsyt.2022.768586

**Published:** 2022-02-28

**Authors:** Michele Ribolsi, Federico Fiori Nastro, Martina Pelle, Caterina Medici, Silvia Sacchetto, Giulia Lisi, Assia Riccioni, Martina Siracusano, Luigi Mazzone, Giorgio Di Lorenzo

**Affiliations:** ^1^Unit of Neurology, Neurophysiology, Neurobiology and Psychiatry, Department of Medicine, University Campus Bio-Medico of Rome, Rome, Italy; ^2^Department of Systems Medicine, University of Rome Tor Vergata, Rome, Italy; ^3^Psychiatry and Clinical Psychology Unit, Fondazione Policlinico Tor Vergata, Rome, Italy; ^4^Department of Mental Health, Azienda Sanitaria Locale (ASL) Roma 1, Rome, Italy; ^5^Child Neurology and Psychiatry Unit, Department of Systems Medicine, University of Rome Tor Vergata, Rome, Italy; ^6^Department of Biomedicine and Prevention, University of Rome Tor Vergata, Rome, Italy; ^7^IRCCS–Fondazione Santa Lucia, Rome, Italy

**Keywords:** autism, psychosis, delusions, hallucinations, negative symptoms

## Abstract

There is strong evidence for the existence of a high comorbidity between autism and psychosis with percentages reaching up to 34. 8% and several significant implications for treatment and prognosis of these patients. However, the identification of comorbid psychosis in patients with Autism Spectrum Disorder represents a complex challenge from a psychopathological point of view, in particular in patients with greater deficits in verbal communication. Intercepting the onset of a psychotic breakdown in autism may be very difficult, both disorders in fact occur along a phenotypic continuum of clinical severity and in many cases, psychotic symptoms are present in an attenuated form. In this paper, we reviewed the available scientific literature about comorbidity between psychosis and autism, focusing our attention on four specific dimensions: delusions, hallucinations, negative symptoms, and clinical course. The aim of this paper is to provide clinical tools to identify these psychotic phenomena in autistic patients, even when they occur in their attenuated form.

## Introduction

If historically schizophrenia and autism were judged to be closely related (1), subsequently, through epidemiological studies, these two syndromes have been reconsidered as two distinct entities, each with its own characteristics, clinical course, and typical onset (2–6). However, there is a growing number of studies that focus their attention on the link between schizophrenia and autism spectrum disorders (ASD), finding significant overlaps in genetic studies, neuroimaging data, clinical signs, and cognitive features (7–10). There is strong evidence for the existence of high rates of comorbidity between autism and psychosis (11). According to literature, up to 34.8% of the patients with a diagnosis of ASD can show psychotic symptoms and, similarly, autistic traits have been reported in schizophrenia patients (SCZ) in a percentage ranging between 3.6 and 60% (12).

Autism spectrum disorder is composed by different levels of complexity, with frequent comorbidity with intellectual disability (13) and recently, there is growing attention on the behavioral equivalents of schizophrenia in people with intellectual disability and autism spectrum disorder (14).

Both ASD and SCZ occur along a phenotypic continuum of clinical severity and are susceptible to common environmental risk factors such as advanced paternal age, pregnancy and birth complications, migration status (12).

Precisely because of this high comorbidity rate between ASD and SCZ, many authors hypothesized the existence of a significant psychotic vulnerability in patients with neurodevelopmental disorders: in particular, impairments in information processing are common in ASD and may favor the risk for a transition to psychosis (12). The expression of autistic core symptoms may vary significantly among ASD patients and may be influenced by the disorder's age of onset. Moreover, psychotic and autistic subthreshold or attenuated symptoms, can make even more difficult to recognize the two comorbid disorders (11).

In this paper, we analyze from a clinical and phenomenological point of view the occurrence of psychosis or attenuated psychotic symptoms in ASD individuals. In particular, we discuss the clinical presentation of four dimensions that must be considered crucial to recognize psychosis in ASD: (1) delusions, (2) hallucinations, (3) negative symptoms, and (4) clinical course of both disorders.

Delusions are defined as “fixed beliefs that are not amenable to change in light of conflicting evidence” (15). According to Jaspers (16), there are three crucial criteria to define delusions: (1) subjective certainty, incomparable to other convictions; (2) imperviousness to counterarguments; (3) implausibility of content. Hallucination represents “the intimate conviction of actually perceiving a sensation for which there is no external object” (17) while negative symptoms are represented by blunted affect, alogia, asociality, anhedonia, and avolition (18).

We chose these three symptoms to identify the presence of psychosis in autism. Negative symptoms have been considered pivotal symptoms of psychosis since the early description of Kraepelin and Bleuler. On the contrary, delusions and hallucinations may theoretically also be present in non-schizophrenic clinical pictures, for example in mood disorders with psychotic manifestations. However, they are an essential element for the detection of the attenuated psychotic syndrome. Finally, the evaluation of the clinical course represents an anamnestic element that allows distinguishing autism complicated by comorbid psychosis from early-onset psychosis or schizophrenia.

In the conclusion section, we suggested some psychopathological elements useful to detect attenuated psychotic symptoms in ASD subjects.

## Methods

A systematic review was conducted based on studies published in English databases from January 1, 1970, to August 1, 2021, following the Preferred Reporting Items for Systematic Reviews and Meta-Analyses (PRISMA) guidelines.

We searched the following English databases: PubMed and SCOPUS.

We used a search strategy based on a combination of the following terms: [(autism) AND (psychosis) OR (schizophrenia) OR (delusions) OR (hallucinations) OR (negative symptoms)] AND [(asperger) AND (psychosis) OR (schizophrenia) OR (delusions) OR (hallucinations) OR (negative symptoms)].

Four independent reviewers screened the literature collected and decided whether a study met the inclusion criteria of the review.

The inclusion criteria were: (1) Time: the systematic review included published articles (without language restrictions) conducted between January 1, 1970, and August 1, 2021; (2) Study participants: we selected papers that studied patients with Schizophrenia Spectrum Disorders and Autism Spectrum Disorders with or without psychotic features; (3) Studies that evaluated the degree of four clinical dimensions: hallucinations, delusions, negative symptoms, and clinical course of both disorders. Theoretical or speculative papers on this topic of high level were included, too.

## Delusions

Delusions are fixed beliefs that are not amenable to change in light of conflicting evidence (15). The false belief is not accounted for by the person's cultural or religious background or his or her level of intelligence (19).

Karl Jaspers in his masterpiece, Allgemeine Psychopathologie (16), proposed three crucial criteria to define delusions: (1) subjective certainty, incomparable to other convictions; (2) imperviousness to counterarguments; (3) implausibility of content. Although it is commonly considered one of the main features of Schizophrenia Spectrum Disorder, delusional beliefs can be recognized in different psychiatric conditions: bipolar disorder, major depressive disorder with psychotic features, neurological and medical disorders like dementia, delirium, or drug intoxication (20), and finally, ASD (21).

### Childish “Fantasies” and Delusional Beliefs in ASD

The presence of delusional beliefs, suspiciousness, and paranoid ideation (22, 23) in autistic children is reported since the early descriptions of Autism and labeled by different authors as schizophrenia-like states (24), borderline conditions (25–27), or severe disturbances of ego development (28). The most common delusional beliefs in ASD individuals are expansive (29), persecutory and passivity delusions, delusion of reference (30) or delusion of thought insertion and withdrawal (31) and “unusual idea” (32).

In some cases, it is not easy to distinguish between “childish fantasies” and delusional beliefs (33). In this regard, Eugene Bleuler in 1950 explained “autistic thinking” as an infantile wish to avoid unsatisfying realities and replace them with imagination and hallucinations. On the contrary, Michael Rutter (34) claimed that ‘the autistic child has a deficiency of fantasy rather than an excess'. So, according to the authors, the word autism may refer to someone who fantasized excessively or someone who did not fantasize at all (35).

Along this line of research, a central question is whether ASD children are able to distinguish between their subjective perceptions and reality. If we assume that the autistic condition implies an intrinsic difficulty in distinguishing between fantasy and reality, the recognition of a delusion in these patients becomes a very complex psychopathological task (36).

### The Role of Language Development in the Onset of Delusions

Most reports about ASD children who develop delusional ideas (30, 37) are characterized by at least two features 1984 (38): a sufficient cognitive level and an adequate communicative ability. These are necessary for the children to express their own thoughts and for the clinician for the evaluation of children's delusions. Unfortunately, the majority of autistic children show an important communication deficit, including mutism and intellectual disability (21). In several cases, it is reported how much may be difficult for these patients to describe their delusions and for the clinicians to identify and classify them (30). For this reason, some authors have hypothesized to investigate the presence of delusional ideas also through drawings (36).

However, according to Jaspers' definition of delusions (16), it is not possible to evaluate this clinical phenomenon in non-verbal subjects. As Jaspers reports, “delusion is communicated in judgments.” “Subjective certainty,” one of the three parameters to define a delusion, can only be assessed verbally. In this sense, the search for delusional phenomena should be carried out only in communicative subjects, with a sufficient cognitive level.

Therefore, most studies have focused on the presence of psychotic symptoms in ASD subjects without intellectual disability and with good language skills (39).

### The Role of Mentalization and Attributional Style in the Onset of Delusions

People with a diagnosis of ASD have difficulties in interpreting subtle social cues, probably because of a Theory of Mind (ToM) deficit, an impairment in the ability to represent the intentions and beliefs of others (40). Both SCZ and ASD subjects frequently present ToM impairments (41–43).

According to Frith (44), ASD children may never acquire ToM abilities, whereas individuals with schizophrenia may have an intact ability to mentalize until their first breakdown. Other authors suggested that, even if impairments in mentalization are clear in both autism and childhood-onset schizophrenia, there are significant differences between the two clinical groups (45, 46). In particular, it has been shown that high-functioning ASD patients are more impaired in affective ToM (verbal faux-pas test) than SCZ subjects (46).

While some studies found no correlation between ToM abilities and the severity of paranoid beliefs (47, 48), other authors have highlighted a possible different quality between the “autistic” paranoia and the “schizophrenic” paranoia. While in ASD subjects, the delusional beliefs may result from the confusion of not understanding the rules and the coordinates of common social interactions (46), in SCZ patients, delusions originate from the aberrant interpretation of others' mental states (44). Compared to ASD, SCZ subjects present a greater hostility bias and paranoid ideation may be considered as the result of hostile attributions (49).

In this regard, several reports have investigated the role of attributional style in the development of paranoia (48). We know that external attributions for negative events and greater internal attributions for positive events may represent a risk factor for the development of paranoia and persecutory delusions (50).

Compared to SCZ subjects, the link between attributional style, paranoid ideation, and mentalization is more controversial in ASD subjects. According to some studies, there is a specific association between (ToM) deficits and paranoia in both ASD and SCZ people (51). An interesting study showed that Asperger subjects have higher levels of paranoia with lower scores on a measure of ToM, supporting the hypothesis that ToM deficits may predispose to persecutory ideas (47). Surprisingly, the authors found no differences in attributional style between ASD individuals and healthy controls (47).

A certain tendency to perceive oneself as an object of social discrimination, potentially vulnerable to judgment, and control by others has been observed in both SCZ patients (52) and Asperger Syndrome patients (47) (see Table 1).

**Table 1:** Key elements for the differential diagnosis of delusions in ASD and Psychosis.

**Autism spectrum disorder**	**Psychosis**
Impairment to understand the rules of common social interactions	Aberrant interpretation of others' mental states
Greater impairment in ToM	Greater tendency to external attributions
Difficulty in distinguish between their subjective perceptions and reality	Hostility bias

## Hallucinations

Hallucination is one of the most relevant symptoms in psychiatry. According to Esquirol, hallucination is “the intimate conviction of actually perceiving a sensation for which there is no external object” (17). Hallucinations, in particular the auditory ones, have been closely linked to schizophrenia (53) and constitute one of the 5 key symptoms of criterion A for the diagnosis of schizophrenia according to DSM-5 (15). Hallucinations have been described also in other psychiatric and medical conditions, drugs or alcohol abuse, and non-clinical people (54). ASD individuals often experience, describe, and exhibit unusual patterns of sensation and, according to DSM 5, sensory anomalies are a core symptom for a diagnosis of ASD (15). However, in clinical practice, it is di?cult to discern between sensory issues presenting in ASD individuals and hallucinations, with significant implications for treatment, prognosis and access to services (55).

### Sensory Behaviors

Findings from standardized parent questionnaires indicate that up to 95% of individuals with ASD show high frequencies of sensory behaviors compared to non-ASD controls (56). With the expression “sensory behavior” we refer to: (a) over-responsivity (exaggerated, rapid onset, and/or prolonged reactions); (b) under-responsivity (unawareness or slow response) to sensory input; (c) seeking behavior, i.e., craving of, and interest in sensory experiences that are prolonged or intense) (57–59).

Interestingly, the frequency of sensory behaviors reported from parents and caregivers seems to vary across age groups and to be linked to mental age.

A meta-analysis from Ben-Sasson (56) shows an increase in sensory behaviors up to age 6–9 years, and a decrease thereafter, compared to non-autistic children. Authors speculate that these symptoms may become more expressed during school age since children typically have to face an increasing demand for independence and the complication of social and physical environment. A true increase of symptoms can, moreover, be reported in the same phase (56). The impact of IQ level on sensory symptoms in autistic people is not clear. Baranek (58) found that sensory symptoms reported by caregivers were negatively related to mental age. A possible explanation is that caregivers may likely underestimate sensory processing problems in children with autism who are less verbally competent (58). In an article by Leekam et al. (60), no significant difference was reported between high functioning and low functioning individuals while other studies have highlighted more total sensory symptoms in younger and lower IQ individuals than older and higher IQ ones (60).

### Anomalous Perceptual Experiences

ASD patients frequently suffer from “anomalous perceptual experiences” (APEs) compared to normal controls (61, 62). For example, perceptions of sounds without a visible source are commonly reported, such as hearing a train 5–10 min before it passes or hearing sounds all around (63, 64).

APEs refer to perceptual and hallucinatory experiences and they are similar to the clinical phenomena commonly associated with psychosis (voices, perceptual distortions, “out-of-body” experiences) (62). Like psychotic patients, ASD patients describe these experiences as intrusive and stressful (62).

Despite being reported also in very young children with ASD (56), first-hand descriptions of APEs have usually been given by high-functioning adults (63, 64), scarce information is available about the nature of perception determining abnormal behaviors in younger and low-functioning subjects.

ASD people reveal that these experiences can be very disturbing or frightening and can impact their daily life and social functioning. Sometimes, ASD individuals are aware that their sensory-perceptual experiences are different from those of non-autistic individuals (64). In this regard, it must be specified that APEs need to be considered psychotic phenomena only if they're interpreted in a delusional way by ASD patients or if their source is attributed to the outside world.

### Confounding Factors in the Recognition of Hallucinations

Describing proper sensory processing might be difficult for autistic patients, mainly because of their deficits in verbal communication and their language impairment (65). As concretism can be present, investigating hallucinations in a person with ASD can result in confounding answers [e.g., “Do you hear voices when no one is there?” “Yes (on the radio)”] (66). What is more, the patients can describe their somatic complaints in unusual ways: a patient who was suffering from headache described his condition saying that “his head was bleeding” (55).

Symptoms misdiagnosed as psychotic, when considered in the context of the developmental disorder, could be better understood as part of it more than the signal of a co-occurring psychosis (55, 67). Given the deficit of language and communication, emotional recognition, social reciprocity, stereotypic interest, theory of mind, and central coherence, Dossetor (67) claims that reliability in this clinical group can be a serious problem in distinguishing true hallucinations from imagination, memories, illusions, and pseudo-hallucinations.

Autistic children reporting “verbal hallucinations,” sometimes have difficulties in recognizing whether they hear their own voice or that of another person and often aren't able to report the specific words or sentences that the voices might be saying (55, 67). For example, a child described auditory hallucinations that sometimes told him to kill himself or other people and that tend to worsen when he felt under stress: a better assessment suggested that he was not truly psychotic but was having pseudo-hallucinations. With therapeutic (not pharmacologic) intervention, he started to recognize that these voices were from his imagination and started to consider them less frightening until they disappeared (67).

For both Van Schalkwyk et al. (55) and Dossetor (67) it is remarkable that these peculiar symptoms in autistic children tend to improve when interventions to manage anxiety and reduce stressful situations are provided, while few or no improvement can be seen when antipsychotic medications are started. On the contrary, authors would consider the existence of a condition of comorbidity when a more abrupt onset of sustained auditory hallucinations and disorganized behavior is noticed and this symptom represents a marked worsening from baseline; moreover, prognosis depends more on the use of antipsychotic medications, than on changes in structure or environmental factors (55).

## Negative Symptoms

Negative symptoms are represented by blunted affect, alogia, asociality, anhedonia, and avolition (18).

They are difficult to assess (68); Andreasen (69–72), they poorly respond to antipsychotics (73, 74), and they are associated with poor functional outcome (75, 76).

Unlike hallucinations and delusions, which are transdiagnostic symptoms, negative symptoms represent a core feature of psychosis and may only be present in schizophrenia or schizoaffective disorder. This is the reason why the history of negative symptoms is sharply related to the history of “Schizophrenia.”

Even before Morel coined the term “Dementia Praecox” (1845), in 1809 John Haslam (77) described a kind of disorder occurring in youth, characterized by “*considerably blunted sensibility*”: “*they do not bear the same affection towards their parents and relations*,” “*they become unfeeling to kindness and careless to reproof*.” Later, Emil Kraepelin (78) identified a broad panel of abnormalities referred to attention, thought, language, volition, and affect that might occur in most but not all patients. Kraepelin's term “*Verblödung”* corresponds to affective “*flattening” or “blunting”* rather than to intellectual dementia (79).

In 1911, Bleuler (1) identified “pathognomonic” symptoms for SCZ, these symptoms have to be present in each patient with SCZ and they are also known as Bleuler 4 As': disturbances of affect, associations, ambivalence, and autism. A Bleuler's student, Eugen Minkowski, introduced the “*loss of contact with reality*” as a key point of schizophrenia and proposed the distinction between two types of schizophrenic autism: “rich or florid autism” (*autisme riche*) and “poor autism” (*autisme pauvre*), which was characterized by affective and cognitive “poverty” (80).

At the same time, in France, Dide and Giraud introduced the concept of Athymhormia, from Greek Thimòs “*the emotion and affect*” and Ormào “*the vital force*” (81). They considered the loss of vitality and affectivity (“*perte de l'élan vital et de l'affectivité*”) as necessary and sufficient elements to characterize schizophrenia.

The dichotomy between positive and negative symptoms kept being enriched thanks to further research proposed by Jackson et al. (82), Crow et al. (83), Crow (84, 85), Andreasen and Olsen (86), and Andreasen (87). Moreover, in the late 1980s, renewing Kraepelin's description of schizophrenia's “fundamental processes,” W.T. Carpenter hypothesized a new refined terminology differentiating negative symptoms in Deficit and Non-Deficit symptoms. Deficit symptoms are primary, they are enduring traits that persist during and between the periods of positive symptoms exacerbation (88). Consequently, Carpenter proposed a new schizophrenia subtype called “Deficit Syndrome” when primary negative symptoms are prominent. Recently a new and revived interest in Negative Symptoms has occurred in order to solve unanswered questions regarding pathophysiology and outcome of schizophrenia (89–91).

### Differential Diagnosis Between Autistic and Negative Symptomatology

Similarities between autism and schizophrenia were recognized since the time of Bleuler and Kanner (92). Only from the early 1970s they have been conceptualized as two distinct disorders (34, 93).

Therefore, differentiating schizophrenia negative symptoms from autistic symptoms represents a pivotal but complicated task even for expert psychiatrists (94, 95).

For example, both social interaction and social communication deficits are frequent in schizophrenia patients as a result of their negative symptoms; at the same time, these are considered key clinical symptoms in ASD (96).

Moreover, the lack of emotional reciprocity in ASD can be confused with the “blunted” affect in schizophrenia.

On one hand, for what concerns “emotional reciprocity,” it is a diagnostic criterion for ASD. Nonetheless, DSM 5 does not precisely explain what reciprocity means, but it only provides anecdotal descriptions such as “*not actively participating in simple social play or games*,” “*preferring solitary activities,”* or “*involving others in activities only as tools or “mechanical” aid”*' (97). ICD-10 illustrated the correspondent diagnostic criteria as follows: “*inadequate appreciation of socio-emotional cues, as shown by a lack of responses to other people's emotions and/or a lack of modulation of behaviour according to social context and, especially, a lack of socio-emotional reciprocity*” (98).

On the other hand, blunted affect is defined as a “*decrease in the expression of emotion and reactivity to events as observed during the spontaneous or elicited expression of emotion (facial and vocal expression and expressive gestures)*” (18) and it manifests itself as a characteristic impoverishment of emotional expression, reactivity, and feeling (86).

According to us, to better differentiate these two concepts, it can be hypothesized that ASD subjects can present “poverty in reciprocity” (which can be similar to the “*decrease in the expression of emotion and reactivity”* of affective flattening) but also an inappropriateness of reciprocity (which is more typical of autism).

Furthermore, also restricted and repetitive behaviors in ASD can be easily misunderstood as a symptom of schizophrenia (99), even if a recent study reported that, in psychosis, a pervasive involvement in special interests or repetitive behaviors is much less common (100).

Restricted and repetitive behaviors might also be present in the co-occurrence of schizophrenia and obsessive-compulsive disorder (OCD); according to a recent review (101) around 30% of patients with SCZ display obsessive-compulsive symptoms (OCSs). What is more, OCSs are considered an identification criterion of an at-risk mental state according to the Comprehensive Assessment of At-Risk Mental State (CAARMS) (102).

Some considerations may help in differentiating these symptoms in Autism and Schizophrenia:

- while in ASD patients, OCSs are present from early childhood (1–3 years of life), in psychotic patients OCSs make their first appearance usually in adolescence (this is the reason why the CAARMS has introduced OCSs in the evaluation of the at-risk mental states).
- while repetitive behaviors in autistic patients are usually a-finalistics, in psychotic patients they may be linked to the prevalent ideation, sometimes of delusional intensity.

## Clinical Course of ASD and SCZ

The clinical course represents a key element for the differential diagnosis between autism and psychosis.

The natural course of SCZ is extremely heterogeneous and generally considered unpredictable (103). The onset of SCZ is usually between the late adolescence and the mid-30s (96) and it follows a prodromal state in which social impairment, atypical interests and unusual beliefs have already occurred. Onset before adolescence is rare (104).

By contrast, ASD is an early onset (12–24 months of age) lifelong condition characterized by persistent deficits in social communication, as well as restricted and repetitive patterns of behavior (96).

Many parents are concerned about affected children's development by age 1, but most of them are worried by age 2 (105). In a minority of cases, about 20%, parents report some period of normal development followed by loss of skills. The clinical significance of regression continues to be debated (106, 107).

A relevant clinical problem is the distinction between autism and childhood-onset schizophrenia (COS) (108).

Childhood-onset schizophrenia (COS) is a rare, chronic mental illness that is diagnosed in children before 13 years of age (109). Children with COS often show premorbid disturbances in social, motor, and language domains with comorbid learning disabilities and mood or anxiety disorders (110).

Additionally, almost one-third of patients meet the criteria for ASD before the onset of COS (111). The outcome of COS is influenced by the presence and severity of these mentioned developmental abnormalities (112) and a few studies suggested that the severity of these deficits may represent a premorbid phenotype for COS (113).

How often a premorbid COS is sufficiently severe to be confused with ASD during childhood?

Diagnostic difficulties are more common in the early phase of these disorders when symptoms are less distinguished.

One of the key difficulties in differentiating COS and ASD is to disentangle true psychotic hallucinations and delusions from the child's imaginative play (114, 115). For example, having imaginary friends is a common situation for children which may be mistaken for psychosis. Similarly, the expressing of children with poor or underdeveloped language skills may be confused with the disorganized thought of SCZ (114).

ASD diagnoses have been reported in COS, in particular we refer to Pervasive Development Disorder Not Otherwise Specified (PDD-NOS) (111) or Multiple Complex Developmental Disorder (MCDD) (99, 116–118).

In a clinical case reported by Hatila and Solanki (119), the patient (an Indian 26 years old male) was diagnosed with ASD (*DSM-IV-TR* criteria) at the age of 4. Then, at the age of 6, he was sent to a special school, from which he was later withdrawn due to aggressive behavior. In adolescence, he presented with unprovoked aggressive behavior, hallucinatory behavior, irrelevant talk and disturbed sleep. A diagnosis of paranoid schizophrenia comorbid with ASD was performed. In this case, there was a history of birth insult, which is underestimated as a possible risk factor for both ASD and SCZ (119).

Keller et al. (120) proposed to operate a differential diagnosis between psychotic disorders and ASD. The key points are the following:

1. the investigation in the early years of some typical traits like interaction and communication difficulties, stereotyped behaviors, selective interests, and the sensorial alteration typical of ASD;
2. the evaluation of the psychotic symptoms within their context of appearance and the search for the cause of these symptoms in a literal reading of the external reality, typical of the ASD;
3. the evaluation of the symptoms typical of schizophrenia, in particular Schneider's first-rank symptoms (e.g., thoughts echoes, sounds coming from thoughts, hearing voices under the form of dialogues and replies to patient questions, hearing voices that accompany the patient's actions, diffusions of the thoughts, delirious perception, and tendency to follow impulses).

## Attenuated Psychosis in ASD Subjects

If the recognition of psychotic symptoms in ASD patients is, by itself, a psychopathological challenge, the detection of attenuated psychotic symptoms in ASD adolescents is an even more complex task, because of the existing overlap between autistic symptoms and subclinical psychotic symptoms.

According to the North American Prodrome Longitudinal Study, 2.6% of young individuals at clinical high risk for psychosis (CHR) present a comorbid developmental disorder (121).

ASD, CHR, and First Episode Psychosis (FEP) share developmental impairments in communication and social functioning, FEP and CHR can be considered in an imaginary midway between healthy subjects and ASD for what concerns the impairments related to social communication and awareness (122).

Furthermore, correlations between autistic symptoms and schizotypal traits (referential thinking, paranoid thinking, constricted affect, social affect, odd speech, eccentric behavior) have been found in individuals with ASD (99, 123). From this perspective, ASD patients will frequently match UHR trait risk factors.

A recent study compared baseline characteristics, clinical profiles, and conversion to psychosis outcomes between young individuals at clinical high risk for psychosis (CHR) who presented with or without a prior ASD diagnosis during the second phase of the North American Prodrome Longitudinal Study (124). However, positive symptoms and all other negative symptoms appear quite similar in individuals with CHR with and without comorbid ASD. Secondly, individuals with ASD do not show a higher risk for converting to psychosis, when compared to other individuals with CHR (124).

Currently, the evaluation of attenuated psychotic symptoms in autistic patients is usually performed using the same tools available for non-ASD patients.

In our opinion, this aspect needs to be discussed more in detail for several clinical reasons.

Firstly, the “level 3” of the “unusual thought of content” dimension of the Scale of Prodromal Symptoms (SOPS) stands for a moderate intensity and it is the “threshold” severity level for an attenuated psychotic symptom. It is described as: “Unanticipated mental events/beliefs that cannot be dismissed and are also irritating and/or worrisome. A sense that unexpected experiences are somehow meaningful because they won't go away.”

It is not easy from a clinical point of view, for a psychiatrist, to understand whether an ASD patient is able to recognize whether a mental event/belief is “unanticipated” or not. Likewise, it is sometimes difficult to define whether, and in what way, a mental event may be meaningful for an ASD patient.

ASD patients may find it difficult to understand their own mental states and to explain whether certain mental events or beliefs are unanticipated or not; they will not easily be able to describe if these mental events or beliefs happen to acquire a more intense meaning, a necessary precondition for the building of a delusion.

In this sense, the extension “tout court” to ASD patients of the classic tools used for the investigation of at risk mental states (SIPS / SOPS, CAARMS) can be controversial.

We must consider the possibility that the investigation of the presence of attenuated psychotic symptoms should be evaluated within a more global assessment of the subject's autistic psychopathology.

An attenuated psychotic symptom cannot be “added” or removed from a patient's symptom framework as if it was an object. As Parnas argues, psychotic symptoms “are subjected to the processes of conceptualization and verbalization, both embedded in the interpersonal context of the psychiatric interview” (125). The interpersonal context between a psychiatrist and an ASD adolescent is very different from the one with healthy help-seeking adolescents.

## Conclusions

The recognition of psychosis in patients with ASD represents a complex psychopathological challenge.

In Table 2, we have summarized the basic differences between ASD and SCZ patients for the detection of delusions, hallucinations, and negative symptoms.

**Table 2:** Basic differences between ASD and SCZ patients for the detection of delusions, hallucinations, and negative symptoms.

**Delusions**	**Not fully evaluable in non-verbal ASD patients**
	**Clinicians should distinguish between “childish fantasies” and clinical delusional beliefs in ASD patients**
	**In ASD patients, delusional ideas have a precise and recognizable time of onset along the clinical course of pre-existing childhood-onset neurodevelopmental disorder**
**Hallucinations**	**ASD patients show more frequently anomalous perceptual experiences (APEs) than true psychotic hallucinations**
	**First-hand descriptions of APEs are usually made only by adult high-functioning ASD patients**
	**APEs should be considered as psychotic hallucinations only if their source is attributed to the outside world**.
**Negative symptoms**	**Unlike autistic symptoms, negative symptoms occur after the psychotic onset and have a progressive worsening clinical course**
	**ASD subjects show “poverty and inappropriateness of reciprocity” rather than affective flattening**
	**Compared to SCZ patients, in ASD loss of social contact Is frequently associated with repetitive behaviors**

The term “psychosis” has various definitions. Generally, this term refers to a “loss of contact from reality” (NHS).

For Minkowski autism is an expression of the loss of vital contact with reality and represents the fundamental psychopathological disturbance in schizophrenia (80).

The modern definition of autism essentially consists of two clinical aspects: (1) persistent deficit in social communication and social interaction and (2) patterns of restricted, repetitive behavior, interests, or activities. Rather than a loss of contact with reality, in ASD patients we observe a loss of *social contact*, or a form of social detachment (126).

Recognizing a “loss of contact with reality” (psychosis) in patients suffering from a form of social detachment (autism) can be very complex.

The correct identification of the three fundamental dimensions of psychosis (Delusion, Hallucinations, Negative Symptoms) and an evaluation of the clinical course of the two disorders may be useful to recognize psychotic comorbidity in patients with ASD.

The identification of delusional, hallucinatory, or negative symptoms, is not easy in patients as complex as patients with ASD.

Recognizing the pathway that leads to a delusion, therefore the moment of its onset, the phases preceding the definition of the delusional construction, etc., may be very complex in ASD patients. While in healthy subjects, the construction of a delusion occurs through a clear rupture with the usual meanings that the patient gives to external reality, in ASD patients it is necessary to recognize this same moment along with a pre-existing impairment in social interaction.

For what concerns the evaluation of psychotic comorbidity in ASD patients, a central issue definitely is the identification of attenuated psychotic symptoms during the transition phase, from adolescence to adulthood. Not surprisingly, there is a growing literature on this topic. To recognize attenuated psychotic symptoms in these patients is essential to prevent or treat earlier the development of psychotic comorbidity.

Foss-Feig et al. (124) show a similar level of core psychotic symptoms in ASD + UHR patients and in the so-called “normotypical” UHR patients. The authors conclude that “baseline psychosis symptoms, predictors of risk for conversion, and ultimate conversion rates are similar in patients with Clinical High Risk with and without ASD.”

In our opinion, these findings need to be confirmed in larger studies.

It would be useful to verify whether there are autistic symptoms within the spectrum, that are associated with a greater risk of conversion to psychosis. The great heterogeneity of the clinical manifestations within the autistic spectrum does not allow universal conclusions to be drawn.

Similarly, the different quality of psychotic symptoms in autistic patients compared to healthy subjects should be studied in depth.

For example, due to the lower hostility bias and the reduced tendency to external attribution of ASD patients compared to psychotic subjects, a lower amount of persecutory delusions should be expected. Likewise, it is necessary to investigate which role concretism plays in ASD patients for what concerns psychotic development.

We hypothesize that the assessment of psychotic risk should be carried out with highly refined diagnostic tools and within the clinical history of the autistic subject.

In this sense, the “tout court” extension of the classic tools for UHR assessment (SIPS/SOPS, CAARMS) deserves further studies.

The tools used so far for the assessment of self-disorders (Examination of Anomalous Self-Experience, EASE) could play a crucial role since they allow the identification of core symptoms of psychosis. A clinical tool, able to recognize the rupture of the ego boundaries and, consequently, the clinical manifestations of the self-disorders within the clinical history of an ASD subject, could certainly be more refined than a tool that allows the identification of an isolated single symptom, for example, an isolated or recurring hallucinatory experience. A psychotic phenomenon, delusional, or hallucinatory, has a more solid clinical value if this is contextualized within the framework of the ego experience disorders. There is still a long way to go.

## Author Contributions

MR, FF, MP, CM, LM, and GD contributed to study the literature on the topic and wrote the paper. GL, MS, and AR contributed to study the literature on the topic and supervised the manuscript. All authors contributed to the article and approved the submitted version.

## Conflict of Interest

The authors declare that the research was conducted in the absence of any commercial or financial relationships that could be construed as a potential conflict of interest.

## Publisher's Note

All claims expressed in this article are solely those of the authors and do not necessarily represent those of their affiliated organizations, or those of the publisher, the editors and the reviewers. Any product that may be evaluated in this article, or claim that may be made by its manufacturer, is not guaranteed or endorsed by the publisher.
